# Novel Metoprolol-Loaded Chitosan-Coated Deformable Liposomes in Thermosensitive In Situ Gels for the Management of Glaucoma: A Repurposing Approach

**DOI:** 10.3390/gels8100635

**Published:** 2022-10-07

**Authors:** Mohamed M. Badran, Abdullah H. Alomrani, Aliyah Almomen, Yousef A. Bin Jardan, Amal El Sayeh Abou El Ela

**Affiliations:** 1 Department of Pharmaceutics, College of Pharmacy, King Saud University, Riyadh 11495, Saudi Arabia; 2 Department of Pharmaceutics, College of Pharmacy, Al-Azhar University, Cairo 11865, Egypt; 3 Nanobiotechnology Unit, College of Pharmacy, King Saud University, Riyadh 11495, Saudi Arabia; 4 Department of Pharmaceutical Chemistry, College of Pharmacy, King Saud University, Riyadh 11495, Saudi Arabia; 5 Department of Pharmaceutics, College of Pharmacy, Assiut University, Assiut 71526, Egypt

**Keywords:** metoprolol, chitosan-coated deformable liposomes, in situ gels, ocular delivery, IOP

## Abstract

Glaucoma is a long-term eye disease associated with high intraocular pressure (IOP), which seriously damages the eyes, causing blindness. For successful therapy, potent drugs and delivery systems are required. Metoprolol (MT) is believed to help reduce elevated IOP. The paradigm of ocular therapeutics may be changed by the integration of chitosan-coated liposomes (CLPs) with thermosensitive in situ gel (ISG). Therefore, MT-CLPs were developed and characterized and compared to uncoated ones (MT-LPs). Furthermore, MT-LP- and MT-CLP-loaded ISGs were prepared and characterized in in vitro, ex vivo, and in vivo studies. MT-LPs and MT-CLPs displayed spherical shapes with nanosize range, reasonable EE%, and significant bioadhesion. The zeta potential changed from negative to positive after CS coating. The extended in vitro drug release of MT-CLPs showed significant mucin mucoadhesion. The formed ISGs were homogeneous with a pH range of 7.34 to 7.08 and a rapid sol–gel transition at physiological temperature. MT-ISG1 (MT-LP) and MT-ISG2 (MT-CLPs-0.5) could increase ocular permeability by 2-fold and 4.4-fold compared to MT-ISG (pure MT). MT-ISG2 demonstrated significantly reduced IOP in rabbits without causing any irritation. In conclusion, MT-ISG2 markedly enhanced corneal permeability and reduced IOP. They would be promising carriers for MT for glaucoma management.

## 1. Introduction

Glaucoma is a chronic eye disease that elevates intraocular pressure (IOP) due to the accumulation and backing of aqueous humor. The increased IOP may cause severe problems, including optic neuropathies, which influence the retina’s neurons and axons. As a result, the accumulated aqueous humor in the eye raises IOP, which in turn affects the nerve cells in the eye, compressing and ultimately killing them, causing blindness [1]. Metoprolol tartrate (MT) is a selective beta-blocker, which is used clinically to treat patients with hypertension. Accordingly, MT can be utilized to treat elevated IOP as an anti-glaucoma medication [2]. Due to first-pass metabolism, MT has a short biological half-life and limited bioavailability when administered orally [3]. Furthermore, local ocular administration of MT-loaded nanocarrier-laden in situ gel (ISG) may be a novel approach to extend the effects on the eye to avoid MT’s systemic side effects when lowering blood pressure. When compared to oral and intravenous administration, ocular medication delivery offers several advantages in terms of safety and targetability. However, due to static and dynamic barriers, the poor drug availability of ocular preparations remains a problem. To overcome these barriers, alternative approaches were researched. Among the potential approaches used to improve ocular absorption of drugs were lipid-based drug delivery systems such as liposomes. It has been reported that liposomes transport the drugs to the eyes more effectively than traditional formulations, yet there are still significant drawbacks. It is still difficult to overcome restrictions including low drug permeability across the cornea, quick drug washout caused by blinking, and high tear turnover rates [4]. Thus, conventional delivery methods have demonstrated lower ocular bioavailability [5]. Based on corneal blockages, which cause a delay in the drug’s ability to reach the eye, there is a greater need for effective ophthalmic preparations [6]. Liposomes have been utilized in the improvement of ocular bioavailability of drugs [7]. They presented biocompatibility and excessive corneal diffusion with an elongated time of residency [8]. However, the limited time taken for conventional liposomes (CLs) to deliver drugs was attributed to their composition [9]. Therefore, deformable liposomes (LPs) were fabricated to cross the ocular barriers efficiently in contrast to CLs due to their high elasticity (9). By changing the surface charge of liposomal systems to a positive charge, improvement attempts were made [10]. Through electrostatic interaction, this strategy is anticipated to lengthen the time when positively charged liposomes are in touch with negatively charged membranes. Another method to extend the period that a medicine is in contact with the tissues of the eye is to use mucoadhesive polymers [10]. It was hypothesized that negatively charged mucin found in the outer layer of ocular tissues might interact with positively charged liposomes electrostatically. This interaction prolongs the resident time by delaying the clearance of drug-loaded liposomes from the eye surface [11]. Liposomes were coated by mucoadhesive polymers to improve the bioadhesion properties of the particles. One of the most effective and extensively utilized mucoadhesive polymers in drug delivery systems is chitosan (CS). Previous investigations showed that the presence of CS could improve the drug transport to the eyes [12]. Due to its high affinity for negatively charged cell membranes (cornea), CS may increase the absorption and therapeutic effectiveness of the drugs [12]. Moreover, CS has a strong potential to open epithelial cell tight junctions, causing an increase in drug diffusion inside the mucosal epithelia of the cornea [13]. However, CS was generally used for modifying liposomes or other nanoparticles [10]. Increasing the viscosity of the ophthalmic preparation is another way to lengthen the residence duration of eye medication. Increased viscosity prevents the drug from being quickly washed out by tears and blinking. Due to their distinct rheological characteristics, in situ gelling agents are typically preferred over other gelling agents when utilized with ocular formulations [14]. These in situ gels (ISGs) typically undergo in situ sol-to-gel transitions as a result of temperature changes, the presence of salts, or changes in the medium’s pH. However, liposomes have a low viscosity, which causes the drug’s post-ocular therapy residence time to be short. Therefore, combining the liposomes with ISG with an increased viscosity is necessary to prevent fast waste [14]. It was shown that there is a phase change from fluid to semisolid gel when exposed to different physiological conditions [15]. Due to the increased temperature, poloxamer 407 has been used in ophthalmic preparations to initiate a sol-to-gel transition. It is frequently used in therapeutic fields to support pre-corneal maintenance in patients [16]. The purpose of this study is to investigate how CS-charged liposomes (MT-CLPs) affect MT delivery to the eye using ISG to increase retention time. To accomplish this, high ocular activity MT-CLPs were created by formulating MT-LPs and coating them with CS. Particle size, zeta potential, encapsulation efficiency, in vitro release, and mucoadhesion were examined. MT-LP- and MT-CLP-laden ISGs were created. Additionally, an ex vivo ocular permeability and a pharmacodynamics investigation were assessed.

## 2. Results and Discussion

It was suggested in the present study that the particle size, positive charge, and lipophilicity of liposomes would promote prolonged pre-corneal residency of the drugs. Furthermore, using in situ gel systems could also increase the residence time because the gel is bioadhesive, greatly enhancing ocular permeability while reducing repeated drug administration. Several studies were conducted to obtain effective formulations.

### 2.1. Particle Size Distribution and Zeta Potential Measurements

The thin-film hydration and electrostatic deposition methods were used to create MT-LPs and MT-CLPs, respectively [17]. Several methods were utilized to characterize the produced preparations to ensure the successful production of MT-loaded cationic LPs. CS is a mucoadhesive cationic polysaccharide that attaches to the mucosa of the eye effectively [18]. As a result, MT-CLPs are intended to keep the formulation on the corneal surface for a prolonged period, leading to an improved ocular effect.

**Figure 1:**
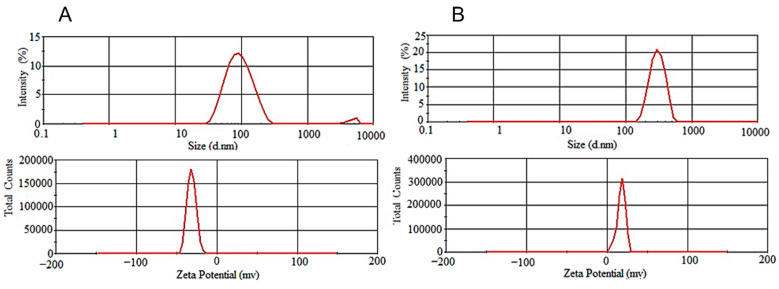
The particle size and zeta potential of MT-LPs (**A**) and MT-CLPs-0.5 (**B**).

The particle size, polydispersity index (PDI), and zeta potential of MT-CLs, MT-LPs, MT-CLPs-0.25, MT-CLPs-0.5, and MT-CLPs-1.0 are shown in Table 1 and Figure 1. The obtained vesicles displayed a nanometer range of particle size. The vesicle size of MT-CLs, MT-LPs, MT-CLPs-0.25, MT-CLPs-0.5, and MT-CLPs-1.0 was 115.2 ± 3.26, 93.3 ± 1.63, 112.2 ± 2.81, 171.1 ± 2.01, and 265.4 ± 4.32 nm, respectively. There was a statistically significant difference between MT-LPs and MT-CLPs (*p* < 0.05). The produced vesicles had a PDI < 0.3, ranging from 0.201 to 0.312, indicating homogeneity and a narrow particle distribution [19]. MT-CLPs-0.25, MT-CLPs-0.5, and MT-CLPs-1.0 showed an increase in their size as the concentration of CS increased. Such an increase in the particle size confirms that CS successfully coated the outer surface of the liposomes [20]. The chemical interactions between the CS hydrogen bond and the lipid head groups may be responsible for this increase [21]. The MT-LPs that include Tween 80 (an edge activator) were shown to have a small particle size. It was previously established that the presence of edge activators in liposomal composition was the most important factor in reducing particle size [22]. Tween 80 has a higher hydrophilic/lipophilic balance (HLB; 15) which could explain this behavior. Tween 80 is known to decrease the surface tension of the medium, which allows the self-assembly of phospholipids in small vesicles [23]. The zeta potential value of uncoated liposomes, MT-CLs, and MT-LPs showed a negative value of −8.2 ± 0.31 and −10.6 ± 0.52, respectively, which agrees with other studies [22]. The high negative value of MT-LPs was attributed to the electronegativity of ethylene oxide moieties of Tween 80 [24]. In contrast, MT-CLPs-0.25, MT-CLPs-0.5, and MT-CLPs-1.0 showed positive values of zeta potential of 17.5 ± 0.49, 25.3 ± 0.97, and 33.9 ± 2.86, respectively. There was a statistically significant difference between MT-LPs and MT-CLPs (*p* < 0.05). CS theoretically possesses a positive charge due to the existence of free amino groups. Therefore, an ionic attraction between these groups and the negatively charged liposome could change the surface charge after coating to a positive value (MT-CLPs). So, the magnitude of the zeta potential indicates the potential coating of the liposomes. The value of zeta potential was increased as the concentration of CS in the formulation increased which could be attributed to the adsorption of an additional layer of CS on the negatively charged liposomes [25]. The shifting of the surface charge from a negative value (MT-LPs) to a positive value after CS coating (MT-CLPs) is an indicator of liposome coating. Alternative mechanisms were presented to illustrate the liposomal coating with CS, such as the electrostatic interaction between anionic liposomal surface and cationic CS [17] and the hydrogen bond between phospholipids and CS [23]. The obtained CS-coated liposomes were confirmed by the increased particle sizes compared to non-coated ones. The positive values of zeta potential were increased along with particle size and PDI based on the concentration of CS. Therefore, any excess of CS could raise the zeta potential values of the liposomes and particle size as a result of the thicker layer produced by the higher CS concentrations. The most ideal coating concentration of liposomes in this study was 0.5% CS (*w/v*), which was chosen because of high EE, significant zeta potential, and acceptable particle size.

### 2.2. Encapsulation Efficiency (EE)

Encapsulation efficiency (EE) is a crucial factor in determining the success of liposomal formulation. The EE of the investigated formulations is presented in Table 1. The EE% of MT-CLs, MT-LPs, MT-CLPs-0.25, MT-CLPs-0.5, and MT-CLPs-1.0 were 13.45 ± 2.51, 26.59 ± 4.88, 27.08 ± 5.19, 27.76 ± 3.09, and 22.05 ± 2.44 %, respectively. The lowest EE% was observed with MT-CLs and MT-LPs. As MT is a hydrophilic molecule, it is expected to concentrate in the aqueous compartment of the liposome. MT was also dissolved in a large amount of water, which was then used to hydrate the dry lipid layer to create liposomes. Moreover, the aqueous core of the liposomes is too small, and the hydration medium cannot be accommodated inside [26].

On the other hand, MT-CLPs showed a slightly increased EE% than that observed with uncoated ones (*p* > 0.05). MT-CLPs-0.25, MT-CLPs-0.5, and MT-CLPs-1.0 showed EE% of 26.59 ± 4.88, 27.76 ± 3.09, and 22.05 ± 2.44 %, respectively. These outcomes revealed that CS coating of liposomes improved the EE% of liposomes, which agreed with a previous study using docetaxel [27] and metronidazole [28] as model drugs. It was suggested that the drug could be held by CS during the coating process, which explains the enhancement in the EE% of the coated liposomes [29]. More CS coating (MT-CLPs-0.5) could drive more drugs on the surface of the liposomes [26]. The slightly increased EE of the coated liposomes suggested that CS had little impact on the EE of the liposomal formulations.

### 2.3. Morphology of MT-LPs and MT-CLPs

**Figure 2:**
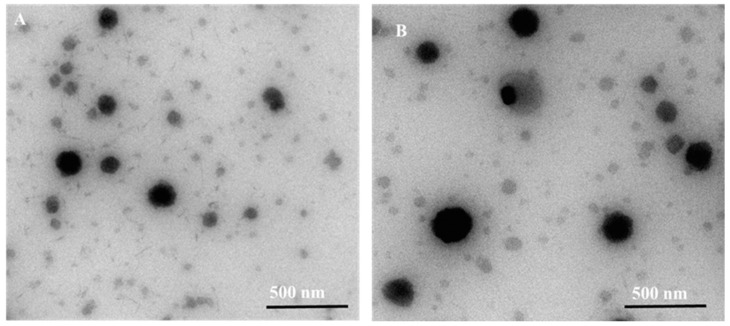
TEM images of MT-LPs (**A**) and MT-CLPs-0.5 (**B**). (MT-LPs: metoprolol loaded deformable liposomes; MT-CLPs: metoprolol loaded chitosan-coated deformable liposomes (0.5% *w/v* CS)).

TEM was used to examine the morphology of the prepared MT-LPs and MT-CLPs-0.5. Figure 2 shows TEM images of MT-LPs with nanosized spherical shapes with uniform monodispersion. Images after coating (MT-CLPs-0.5) indicated a slightly larger particle size. The particle size of MT-LPs and MT-CLPs-0.5 was about 90 and 170 nm, which was in good correlation with results obtained by particle size measurement. Thus, these images matched the outcomes identified by the dynamic laser light scattering method. The findings confirmed that CS might successfully coat the surface of MT-CLPs-0.25. Therefore, the formation of a CS film layer on the outer surface of the liposomal particles is evidenced by the change in the shape of MT-CLPs-0.5.

### 2.4. Physical Stability Study

The particle size measurement was used to inspect the physical stability of MT-LPs and MT-CLPs. After one month of storage at 4 °C, the prepared MT-LPs, MT-CLPs-0.25, and MT-CLPs-0.5 showed minor variations in particle size (data not shown). The presence of a CS layer over the liposomes was expected to keep the vesicles physically stable [23].

### 2.5. In Vitro MT Release Study

It has been revealed that the residence time of the carriers in vivo can affect drug therapy. Therefore, in vitro release profiles may predict the in vivo behavior of a drug carrier [30]. All of the coated liposomes displayed slower release patterns than the uncoated ones, confirming the coating formation.

**Figure 3:**
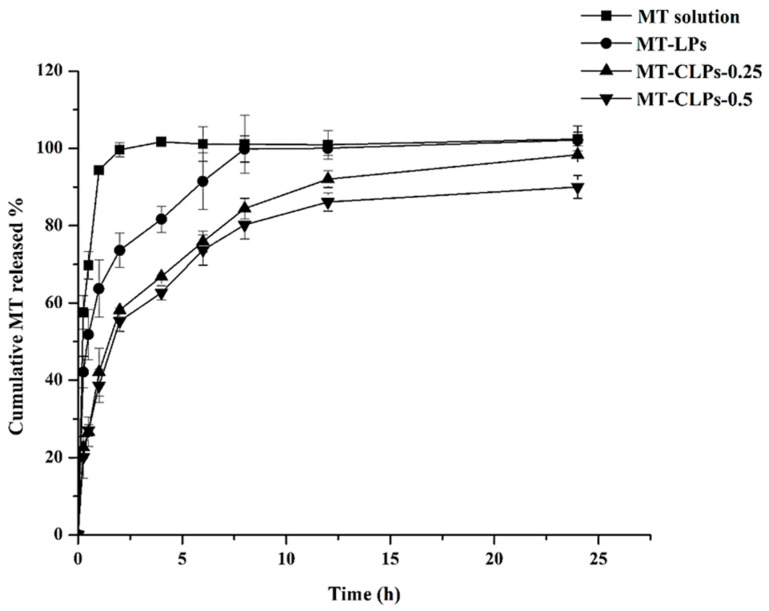
In vitro release profiles of MT-LPs, MT-CLPs-0.25, and MT-CLPs-0.5 (mean ± SD, *n* = 3).

The in vitro release of MT from MT-LPs, MT-CLPs-0.25, and MT-CLPs-0.5 was studied and compared to MT solution (control) (Figure 3). In general, the aqueous solution of MT showed a fast and complete release of MT in comparison with the prepared systems. For MT solution, almost all of the drug was released (100%) within the first 2 h, whereas 73.63 ± 4.51%, 58.41 ± 2.51, and 55.29 ± 2.62 of MT were released from MT-LPs, MT-CLPs-0.25, and MT-CLPs-0.5 within the same time. This rate of MT release from the solution form is expected due to the hydrophilic nature of MT [13]. The percentages of MT release from MT-LPs, MT-CLPs-0.25, and MT-CLPs-0.5 systems were 92%, 76%, and 74% after 6 h and 100.1% ± 1.9, 92% ± 2, and 86% ± 2, respectively, after 12 h. The high initial release of MT from the MT-LP system could be attributed to the leaching of un-entrapped MT adsorbed on the outer surface of the carriers. The hydrophilicity of MT and flexibility of the liposomal membrane due to the presence of Tween 80 in its composition [23] could facilitate the leaking out of MT from the lipid membrane. The sustained release behavior of MT-CLPs-0.25 and MT-CLPs-0.5 could be attributed to the layer of CS that covers the outer surface of the liposomes, which hampers the diffusion of MT, causing a slow release of MT from the lipid matrix [23].

The in vitro release kinetic model of MT from the formulated systems was estimated using different mathematical models, comprising the zero-order, first-order, Higuchi, and Korsmeyer–Peppas kinetic models, as shown in Table 2. The correlation coefficient (R^2^) value was used to figure out the release kinetic model of MT, in which the mathematical model that has the highest R^2^ value is most likely to represent the release kinetic model. Accordingly, the data presented in Table 2 revealed that the release model of MT from MT-LPs, MT-CLPs-0.25, and MT-CLPs-0.5 fit with Korsmeyer–Peppas’s equation. As shown, the *n* value (release exponent) is used to characterize different release mechanisms. It was found that *n* values are mostly less than 0.43, suggesting the release mechanism was governed by diffusion. This behavior implies that the drug released from the system follows a Fickian transport pattern.

### 2.6. Mucoadhesive Study or Bioadhesion

**Figure 4:**
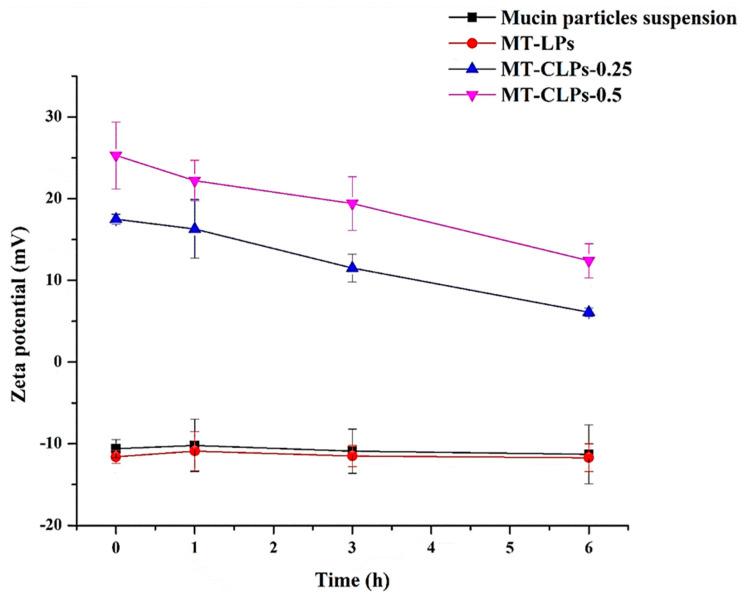
The zeta potential values of MT-LPs, MT-CLPs-0.25, and MT-CLPs-0.5 during incubation in 0.1% mucin dispersion (mean ± SD, *n* = 3).

The mucoadhesive properties of the prepared systems were evaluated using the mucin particle method. The investigated formulations were placed in a dispersion system containing mucin particles and the zeta potential of dispersed particles was evaluated after 1, 3, and 6 h of incubation using 0.1% mucin dispersion as a control (Figure 4). The zeta potential of the dispersion containing 0.1% mucin and MT-LPs was negative and no significant change was observed after 1, 3, and 6 h of incubation. On the other hand, the zeta potential of the MT-CLPs changed gradually when they were incubated with 0.1% mucin for up to 6 h. CS coating significantly changed the surface characteristics of mucin particles. After being incubated with mucin for around 6 h, the zeta potential of mucin particles mixed with MT-CLPs-0.25 and MT-CLPs-0.5 changed to positive values of 6.1 ± 0.5 and 12.4 ± 2.4, respectively. The electrostatic interaction between positively charged CS-coated LPs and negatively charged mucin particles could explain this change. It could be concluded from the significant change in the zeta potential that MT-CLPs-0.25 and MT-CLPs-0.5 have a high affinity to interact with mucin. Such interaction helps the enhancing of bioadhesion property of the CS-coated liposomes [31]. Accordingly, the positively charged carriers could interact with cell mucosa, which encourages long-term adhesion and retention of MT, improving its therapeutic result [23,31].

### 2.7. Characterization of the Thermosensitive ISG

A mixture of poloxamer 407 and HPMC was used as an in situ gelling agent for MT-LPs (MT-ISG1) and MT-CLPs-0.5 (MT-ISG2). These systems were successfully prepared and characterized in terms of pH, gelation temperature, viscosity, mucoadhesion properties, and ex vivo transcorneal permeation. The pH of MT-ISG1 and MT-ISG2 was within the range of 6.5–7.4, which is close to the pH range of tear fluids of 7.34–7.08. The eye is sensitive to pH, so it is important to control the pH of ocular formulations to avoid unpleasant side effects such as eye irritation.

#### 2.7.1. Determination of the Sol–Gel Transition Temperature

One of the crucial factors in the formulation of thermosensitive gel is the gelation temperature [32]. To provide precise dosing, the preparation for thermosensitive ISG must remain liquid at room temperature [33]. The liquid dispersion of MT-ISG1 and MT-ISG2 appeared clear at room temperature by visual examination. It has been reported that the ocular mucosal layer has an average temperature of 34 °C [32]. Thus, ISG1 and ISG2 displayed sol–gel transition at 34 °C after dilution with STF at a ratio of 25:7 to mimic the conditions of the human eye. This indicates that MT-ISG1 and MT-ISG2 might form a viscoelastic gel when it is in contact with the eye environment in the presence of tear fluids.

#### 2.7.2. Viscosity Measurement

The transformation of ISGs from the form of a solution at room temperature into a gel upon interaction with biological conditions is one of their favored aspects. The ocular application of the drug in solution form will help in ensuring dose accuracy. Therefore, the viscosity of ISG at a storage temperature and physiological temperature of the eye should thus be measured. Here, the formulations’ viscosity was determined at 25 and 34 °C. The success of ISGs for ocular delivery is attributable to their characteristics after delivery, and how fast sol–gel transition took place after being delivered into the eye. The viscosity alteration of MT-ISG1 and MT-ISG2, as a result of the change in the environment temperature, was evaluated. The formulations, MT-ISG1 and MT-ISG2, were in a liquid state at 25 °C with viscosity values of 573 and 815 cP, respectively. It has been observed that liquids with low viscosity are suited for ocular use. When the temperature of the environment increased to 34 °C, MT-ISG1 and MT-ISG2 transformed quickly into a gel state and reported 16,833 and 23,442 cP, respectively. The gelation stimuli (increasing the temperature to 34 °C) were utilized to facilitate ocular drug administration [34]. It was noticed that MT-ISG2 (containing MT-CLPs-0.5) reported a higher viscosity value than MT-ISG1 (containing MT-LPs). High-viscosity formulations are anticipated to prolong drug release and reduce treatment frequency. A viscosity of up to 3500 cP (at 25 °C) has been suggested for ease of application [35].

#### 2.7.3. Ex Vivo Transcorneal Permeation Study

**Figure 5:**
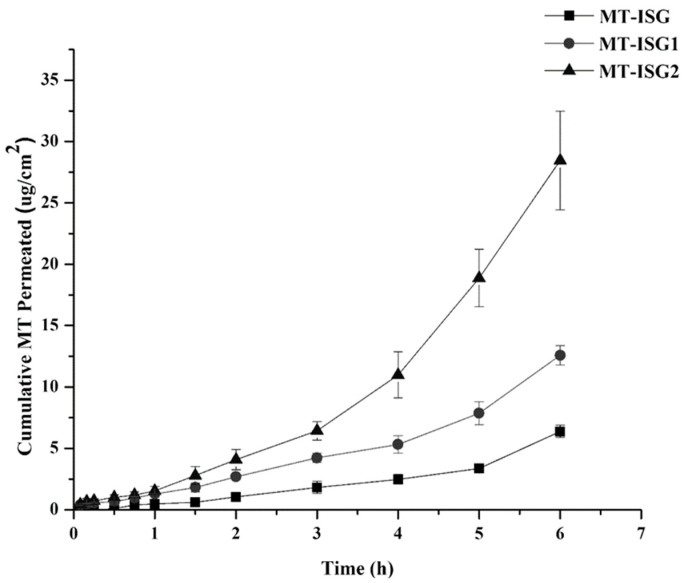
The transcorneal permeation profiles of MT-ISG, MT-ISG1, and MT-ISG2 (mean ± SD, *n* = 3).

Improved therapeutic efficacy of MT following ocular application is the main objective of adding MT to a thermosensitive ISG as an ocular preparation. In order to investigate this aim, an excised goat eye model was used to study the transcorneal permeation of MT. Figure 5 displays the permeation profiles of MT-ISG, MT-ISG1, and MT-ISG2. According to this graph, 6.4, 12.6, and 28.5 µg/cm^2^ of MT cumulatively permeated through the cornea after 6 h of administration of MT-ISG, MT-ISG1, and MT-ISG2, respectively.

It has been detected that MT-ISG exhibited the least quantity of MT permeated. In comparison to the control sample (MT-ISG), the presence of MT in nanocarrier systems greatly increased its transcorneal permeation by about 2-fold (MT-ISG1) and 4.4-fold (MT-ISG2). Additionally, MT-ISG2 (coated liposomes) enhanced transcorneal MT permeation by 2.4-fold compared to an uncoated liposomal formulation (MT-ISG1).

This behavior reveals the beneficial effect of the CS layer that coats the liposomes’ outer surface. The distribution of the incorporated drugs is facilitated by the prepared nanocarriers with diameters less than 10 µm diffusing efficiently across the cornea [36]. The improved permeation of MT may be explained by the nanoscale particle size of the produced MT-LPs and MT-CLPs-0.5. The flexible membrane of the deformable liposomes (presence of Tween 80) may be the cause of the higher MT-LP corneal permeability [35]. Additionally, MT-CLPs-0.5 showed a higher permeation of MT than MT-LPs due to electrostatic interaction and hydrogen bonding [13,23]. In addition, it has been reported that CS-coated nanocarriers could increase the cornea’s permeability by opening intracellular or intercellular tight junctions between corneal epithelial cells [23]. Then, the MT-ISG2 formulation is expected to increase MT ocular bioavailability after application depending on the results of the ex vivo transcorneal permeation study.

#### 2.7.4. Pharmacodynamic Study

**Figure 6:**
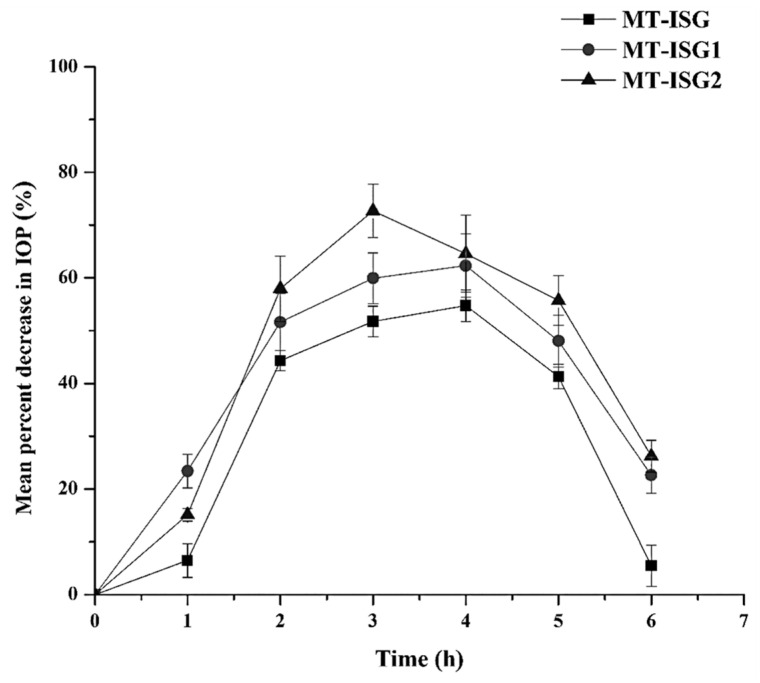
The mean percent decrease in IOP–time profiles following a single ocular dose of MT-ISG, MT-ISG1, and MT-ISG2 in rabbit eyes (mean ± SD, *n* = 3).

MT as a beta-blocker is well recognized to lower IOP. The in vivo pharmacodynamics of MT-ISG, MT-ISG1, and MT-ISG2 containing 0.5 % (*w/v*) MT were evaluated using rabbits as animal models (Figure 6 and Table 3). Following a single ocular dose, Figure 6 displays the mean percent decrease in IOP–time profiles, and Table 3 displays the mean pharmacodynamic parameters. The IOP reduction effect of MT was not observed clearly after 1 h of MT-ISG instillation, while the reduction in IOP was observed at 2, 3, 4, and 5 h post-application. On the other hand, MT-ISG1 and MT-ISG2 showed a reduction in IOP from the first h of application, and their effect continued longer than that observed with MT-ISG. This is in accordance with the reported data by Abou El Ela et al. (2014) who concluded that MT ophthalmic gels extended the ocular contact time for more than 5 h [14,37]. After 6 h, MT-ISG2 displayed a (percent Dec IOP)_max_ of 73.6 ± 4.13%. When compared to MT-ISG1 and MT-ISG, which demonstrated a (percent Dec IOP)_max_ of 62.3 ± 6.28% and 54.7 ± 3.15%, respectively (*p* < 0.05), this (percent Dec IOP)_max_ was much greater. IOP MT-ISG showed a decrease of 5.5 ± 1.19% after 6 h, while MT-ISG1 and MT-ISG2 showed a decrease of 22.6 ± 3.44% and 26.2 ± 3.11%, respectively. This suggests that MT-ISG2 consistently delivered MT for a considerable period. This prolonged effect on IOP reduction resulted from increased ocular permeation of MT upon application of MT-ISG2 due to increased drug retention and contact time [38]. In comparison to MT-ISG (2.3 h), MT-ISG1 and MT-ISG2 demonstrated a considerably longer MRT of 5.7 and 6.2 h, respectively (*p* < 0.05). The nanocarriers incorporating ISG exhibited longer drug release and sustained lower IOP based on the higher drug retention at the site of action. In terms of T_max_, MT-ISG, MT-ISG1, and MT-ISG2 demonstrated non-significant T_maxes_ of 4, 4, and 3 h, respectively. Then, MT-ISG2 reached its maximal effect after 3 h of administration. This elevation in MRT of MT-ISG2 could be clarified by increasing contact time with the corneal tissues. The behavior could be attributed to the positively charged CS, which yields greater drug absorption and consequently increased MRT. The ability of MT-ISG1 and MT-ISG2 to decrease IOP was further demonstrated by the fact that their AUC_(0–6)_ was significantly higher than that of MT-ISG (*p* < 0.05). MT-ISG1 and MT-ISG2 had AUC_(0–6)_ values of 256.5 ± 19.26% h and 279.1 ± 27.83% h, respectively. Furthermore, MT-ISG had AUC_(0–6)_ values of 199.2 ± 11.73% h. Increases of 1.28- and 1.41-fold in AUC_(0–6)_ following the application of MT-ISG1 and MT-ISG2 were observed compared to MT-ISG. It is concluded that MT-ISG2 produces a considerable effect on the IOP. Therefore, MT-ISG2 was a promising carrier for MT. This positive impact could be attributed to the CS coating playing a significant role in controlling the drug release, prolonging the ocular contact time, and promoting ocular permeation. 

#### 2.7.5. Eye Irritation Study

Using a modified Draize’s test on a rabbit as the model animal, the irritant potential of MT-ISG, MT-ISG1, and MT-ISG2 was examined (Table 4). Large eyes, ease of handling, low cost, and availability were all factors in the choice of rabbits. Compared to human eyes, rabbit eyes are more susceptible to irritating compounds [39]. The obtained formulations had a mean score of 0 and were determined to be non-irritating (data not shown). However, the MT-ISG2 formulation caused conjunctival redness after 1 h of instillation, which disappeared after 3 h to return to normal. The cationic characteristics of CS may be the cause of the transient discomfort caused by MT-ISG2. Up to 24 h after treatment, no abnormal symptoms were seen after ocular application.

## 3. Conclusions

MT-LPs, MT-CLPs-0.25, MT-CLPs-0.5, and MT-CLPs-1.0 were successfully established as spherical shapes with nanosize, low EE, and positively charged zeta potential values (MT-CLPs). The findings demonstrated the mucoadhesive property of MT-CLPs-0.25 and MT-CLPs-0.5. based on zeta potential measuring before and after mixing with mucin. MT-CLPs-0.25 and MT-CLPs-0.5 released MT continuously for 24 h, according to the in vitro release investigation. A sol–gel transition after the instillation of ISG into the eye demonstrated that ISG had the desired viscosity under physiologic conditions. As evidenced by enhanced permeation and prolonged pre-corneal retention, MT-ISG2 displayed effective carriers for ophthalmic usage. MT-ISG2 confirmed a considerable reduction in IOP when compared to MT-ISG and MT-ISG1. The appropriateness and safety of the produced MT-ISG1 and MT-ISG2 for human use were demonstrated by investigations of in vivo ocular irritation. These results demonstrate the potential use of MT-ISG2 (MT-CLPs-0.5) for improving pharmacodynamic efficacy after ocular administration.

## 4. Materials

Metoprolol tartrate (MT) was kindly provided by Sid. Co., for Pharmaceutical and Chemical Industry (Cairo, Egypt). Lipoid S100 (phosphatidylcholine (PC) that contains approximately 94% soybean lecithin was purchased from Lipoid GmbH (Ludwigshafen, Germany). Cholesterol (Chol) and chitosan (CS) were obtained from Sigma-Aldrich Chemical Co. Ltd. (St. Louis, MO, USA). Methanol and acetonitrile (HPLC grade) were obtained from Fisher Scientific Co. (Hampton, NH, USA). Tween 80 was purchased from BDH, Organics (England, UK). All other reagents were of analytical grade.

## 5. Experimental

### 5.1. Preparation of MT-LPs and MT-CLPs

Using a thin-film hydration method similar to that described previously [27], MT-LPs were prepared with some modifications. MT-LPs were composed of PC, Chol, and Tween 80, while MT-loaded conventional liposomes (MT-CLs) containing PC and Chol were prepared for comparison. Briefly, PC, Chol, and Tween 80 in the molar ratio of 0.9, 0.3, and 0.1 M, respectively, were dissolved in a 10 mL chloroform:methanol mixture (2:1 *v/v*). The organic solvents were evaporated using a rotary evaporator (Buchi Rotavapor R200, Buchi Architect Co., Ltd., Flawil, Switzerland) for 4 h at 40 °C until dried film lipid was achieved. The resultant film was flushed with nitrogen gas to ensure that all organic solvents had been removed completely. Rehydrating of the dried film was accomplished with 10 mL of phosphate buffer (pH 7.4) containing MT under vortexing for 30 min. MT-LPs dispersions were sonicated in a water bath for 10 min to remove the residual lipid film followed by probe-sonication for 3 min at 60% amplitude to form a nanorange of MT-LPs. 

For CS-coated MT-LPs (MT-CLPs), an equivalent volume of MT-LPs was added dropwise to CS solutions (0.5%, 1%, and 2% *w/v*) under probe-sonication for 3 min [27]. CS solution was previously dissolved in acetic acid solution (0.1% *v/v*). The resulting suspension was magnetically stirred for 2 h at room temperature to obtain MT-CLPs. Several CS-coated liposomes were obtained containing 0.25% *w/v* CS (MT-CLPs-0.25), 0.50 % *w/v* CS (MT-CLPs-0.25), and 1.0 % *w/v* CS (MT-CLPs-1.0). To ensure the quality of all formulations, size, polydispersity index (PDI), zeta potential, and encapsulation efficiency (EE) were evaluated. The obtained dispersions were stabilized using a freeze dryer (Alpha 1–4 LD Plus, Martin Christefriertrocknugsanlagen GmbH, Osterode am Harz, Germany) at −60 °C for 72 h. All formulations were employed in triplicate.

### 5.2. Characterization of MT-LPs and MT-CLPs

#### 5.2.1. Particle Size Distribution and Zeta Potential Measurements

The average particle size distribution and zeta potential were assessed based on dynamic light scattering (DLS) and electrophoretic mobility, respectively, using the Zetasizer Nano ZS (Malvern Instruments, Malvern, UK) at 25 °C. Before the measurement, the formulations were diluted with filtered deionized water (1:100) to avoid the phenomenon of multiple scattering. For each sample, the results were averaged from triplicates.

#### 5.2.2. Determination of Encapsulation Efficiency 

Encapsulation efficiency (EE%) of MT-CLs, MT-LPs, and MT-CLPs was indirectly determined by ultracentrifugation [40]. The obtained dispersions were centrifuged at 40,000 rpm for 30 min using Optima™ MaxE, a super-cooled centrifuge (Beckman Coulter, Pasadena, CA, USA). A supernatant containing the unencapsulated drug (MT_free_) was collected and spectrophotometrically measured at λ_max_ at 275 nm [41]. Experiments were carried out in triplicate. EE% was calculated using the following equation:

(1) $$\mathrm{EE}\%\;=\frac{{\mathrm{MT}}_{\mathrm{total}}\;-\;{\mathrm{MT}}_{\mathrm{free}}}{{\mathrm{MT}}_{\mathrm{total}}}\;\times \;100$$

where MT_total_ and MT_free_ represent the total drug added and the unencapsulated drug, respectively.

MT-LPs, MT-CLPs-0.25, and MT-CLPs-0.5 were selected for further experiments based on the findings of particle size analysis, zeta potential, and EE%.

#### 5.2.3. Morphology of MT-LPs and MT-CLPs

The morphology of MT-LPs and MT-CLPs-0.5 was observed with a transmission electron microscope (TEM) at 60 kV (JEM1011, JEOL, Tokyo, Japan). After suitable dilution, the sample was negatively stained with a 1% (*w/v*) aqueous solution of uranyl acetate on a copper grid. Filter paper was used to remove the extra liquid from the grid, which was then air-dried at ambient temperature. The sample was then examined under a TEM.

#### 5.2.4. Physical Storage Stability

At 4 °C, the physical storage stability of MT-LPs, MT-CLPs-0.25, and MT-CLPs-0.5 was tested for one month. After storage, the particle size and PDI were calculated. The experiment was carried out three times.

#### 5.2.5. In Vitro Release Study

In vitro release patterns of MT solution, MT-LPs, MT-CLPs-0.25, and MT-CLPs-0.5 were investigated in PBS (pH 7.4) employing the dialysis bag method [42]. In pre-swelled dialysis bags (molecular weight cutoff 12,000–14,000), 1.0 mL samples were sealed. Then, the sealed bags were soaked in 50.0 mL PBS (pH 7.4) under a magnetic stirrer (100 rpm) at 37 ± 0.5 °C. At pre-defined time intervals (0.5, 1, 2, 4, 6, 8, 12, and 24 h), 2.0 mL samples were taken and replaced with 2 mL of fresh release medium. After 0.22 um filtration, the collected samples were measured using a UV spectrophotometer set to 275 nm. The study was performed in triplicate. Kinetic analysis of the in vitro release data was carried out after collected data had been fitted to various kinetic models, including zero-order, first-order, Higuchi, and Korsmeyer–Peppas.

#### 5.2.6. Mucoadhesion Study

Based on the interaction of mucin with the MT-LPs, MT-CLPs-0.25, and MT-CLPs-0.5, the mucin particle technique was used to measure mucoadhesion [31,43]. By measuring zeta potential changes after the interaction of the investigated samples with the negatively charged mucin, the bioadhesion properties were identified [31]. At a concentration of 10 mg/100 mL, bovine mucin powder was suspended in PBS (pH 7.4). In comparison to pure mucin particle suspension, a known weight (50 mg) of each formulation was added to a 3 mL mucin suspension and blended by vortexing at room temperature for 1, 3, and 6 h of incubation. After that, the change in the zeta potential value of the mucin suspension or mucin-containing formulation was described as an index of mucoadhesion.

MT-LPs and MT-CLPs-0.5 were selected for further experiments based on the results of in vitro drug release and mucoadhesion studies.

### 5.3. Preparation of MT-LPs and MT-CLPs-0.5 Loaded In Situ Gel

A mixture of poloxamer 407 and HPMC was used to produce thermosensitive in situ gel (ISG) containing MT-LPs (MT-ISG1) and MT-CLPs-0.5 (MT-ISG2), equivalent to 0.5% *w/v* of MT. Cold water was used to dissolve HPMC (0.5% *w/v*) under slow agitation to obtain a clear solution. Then, poloxamer 407 was added to the HPMC solution followed by refrigeration under stirring to complete the hydration and swelling of the polymers. The obtained ISG was kept in the refrigerator (8 °C) overnight. For the viscosity modifier, HPMC was used. In addition, pure MT-loaded ISG (MT-ISG) was prepared in cold water for comparative analysis. The produced ISG formulations were kept at 4 °C until further evaluation.

#### 5.3.1. Characterization of the Thermosensitive ISGs

The developed ISG formulations were visually inspected under light against a white and black background to determine the clarity of the formulations before and after gelation. A calibrated digital pH meter was used to determine the pH of MT-ISG1 and MT-ISG2 at room temperature. The pH tests were conducted in triplicate.

#### 5.3.2. Determination of the Sol–Gel Transition Temperature

To determine the sol–gel transition temperature, 2 mL of ISG was poured into a closed test tube vial and placed in a thermostat-regulated bath shaker (JULABO GmbH, Seelbach, Germany). The water bath’s temperature was raised from 20 to 40 °C progressively in 0.5 °C increments. To confirm gel formation, the vials were tilted 90° at intervals of 1 °C [23]. The temperature at which the gel did not flow while the vial was being tilted was identified as the sol–gel. The evaluation was done in triplicate.

#### 5.3.3. Viscosity Measurement

The viscosity of the ISG samples was measured using a Sine-wave Vibro viscometer, SV-10 Series, made by A&D Company, Ltd. (Tokyo, Japan). Viscosity measurement for all samples was carried out at a constant shear rate (100 s^−1^) and at a temperature of 25 °C and 34 °C to study the ISG’s thermo-gelling behavior.

#### 5.3.4. Ex Vivo Transcorneal Permeation Study

The ex vivo corneal permeation protocol was approved by the Research Centre Ethics of King Saud University, College of Pharmacy, Riyadh, Saudi Arabia (Ref. No.: KSU-SE-22-23). The permeation of MT-ISG, MT-ISG1, and MT-ISG2 through the corneal membrane was investigated using a goat cornea. The goat’s eyeball was attained from a slaughterhouse and moved to the lab where it was placed in normal physiological saline kept at 4 °C. The cornea and surrounding 5–6 mm scleral tissue were gently removed and then cleaned with cold saline. The cleaned cornea was preserved in a freshly prepared cold tear buffer of pH 7.4. Franz’s vertical diffusion cell was used to investigate the ex vivo transcorneal permeation. Specific amounts of MT-ISG, MT-ISG1, and MT-ISG2 (corresponding to 0.5% MT) were applied to the corneal surface connected to the acceptor medium (freshly prepared PBS, pH 7.4) at 100 rpm and 37 ± 0.5 °C. At intervals of 5, 15, 30, 45, 60, 120, 180, 240, 300, and 360 min, aliquots of 1 mL were taken out and replaced with the same volume of a buffered solution. There were three runs of each experiment. The permeated amount of MT was analyzed by a previously validated HPLC method with slight modification [44]. HPLC with a Waters™ system (Waters™, Milford, MA, USA) was utilized by injection of the sample (20 μL) into the system under isocratic elution using a mixture of phosphate buffer (pH 3, containing 0.5% triethylamine):methanol:acetonitrile (90:1:9) as mobile phase. The flow rate was 1.2 mL/min through a reversed-phase C18 column (Bondapak™, 4.6 × 150 mm, 10 mm particle size). The eluted samples were detected by a UV detector set at 275 nm. All procedures were carried out at room temperature.

#### 5.3.5. Pharmacodynamic Study

The in vivo response was evaluated using the effect of MT on intraocular pressure (IOP) following the application of MT-ISG, MT-ISG1, and MT-ISG2. MT has been demonstrated to lower IOP when administered topically to the eyes [45]. The pharmacodynamic study protocol was approved by the Research Centre Ethics of King Saud University, College of Pharmacy, Riyadh, Saudi Arabia (Ref. No.: KSU-SE-22-23). Nine adult male albino rabbits weighing 2.5–3.0 kg were used. During the testing, the collected animals were held upright in their separate restraint cages. To anesthetize the cornea, isotonic xylocaine solution (2% *w/v*) was added to the rabbits’ eyes. The diameter of the pupil or the IOP of the eye was unaffected by xylocaine [46].

MT-ISG, MT-ISG1, and MT-ISG2 were administered topically in doses of 50 µL each. The doses were positioned in the right eye’s lower conjunctival sac, while the opposite eye served as a control. The rabbits were positioned in a supine position after receiving one drop of the xylocaine solution used as a surface anesthetic. To allow the drug to contact the eyeball, the lower eyelid was pulled back and the upper eyelid was slightly lifted. Following the administration of the test substance, the eyelids were held for 30–40 s while the IOP of both eyes was measured, beginning with the right. The IOP was measured using the Schiotz Tonometer (Rudolf Riester GmbH and Co. KG, Germany) at the following time intervals: 0 (pre-dose), 1, 2, 3, 4, 5, and 6 h after each application. 

The average IOP was determined after three measurements. The percent reduction in IOP at each time point was determined using Equation (1) [47].

(2) $$\;\mathrm{reduction}\;\mathrm{in}\;\mathrm{IOP}=\frac{{\mathrm{IOP}}_{\mathrm{control}}\;-\;{\mathrm{IOP}}_{\mathrm{treated}}}{{\mathrm{IOP}}_{\mathrm{control}}}\;\times \;100$$

Various pharmacodynamic parameters based on the % reduction in IOP–time data were determined. The % reduction in IOP ((% IOP)_max_) and the time to reach the maximum % reduction in IOP (t_max_), the area under the curve (AUC_(0–6)_, %.h), and the mean residence time (MRT) were calculated. 

#### 5.3.6. Eye Irritation Study

Under in vivo conditions, a modified Draize test was utilized to examine the eye irritation of the selected formulations [48,49]. Four adult male New Zealand albino rabbits weighing about 2–3 kg were used. The rabbits were in good health and kept in regular conditions of humidity, light, air, and temperature with standard meals and water. Irritation tests were performed on MT-ISG, MT-ISG1, and MT-ISG2 using lighting. Each rabbit had 50 µL of each sample instilled directly into the lower right cul-de-sac, with the opposite eye serving as a control. A saline solution (0.9% NaCL) was used as the control... The ocular irritation after 0, 1, 3, 6, and 12 h was evaluated using an assessment scale adopted from a previous study [21]. A scoring scale of 0–3 was used to rank the conjunctival discharge, conjunctival chemosis, and conjunctival redness; 0 is assigned for normal conditions, and 3 for extreme irritation.

#### 5.3.7. Statistical Data Analysis

The Origin software, version 8, and Microsoft Excel, version 2010, were both used for data analysis. One-way analysis of variance (ANOVA) was used to examine the statistical differences between groups. Values of *p* < 0.05 were considered statistically significant. The mean ± standard deviation is used to express the results.

## Figures and Tables

**Table 1:** The characterization of the different types of liposomes (*n* = 3, mean ± SD).

Formulations	Particle Size (nm)	PDI	Zeta Potential (mV)	EE%
MT-CLs	115.2 ± 3.26	0.201 ± 0.001	−8.2 ± 0.31	13.45 ± 2.51
MT-LPs	93.3 ± 1.63	0.243 ± 0.005	−10.6 ± 0.52	26.59 ± 4.88
MT-CLPs-0.25	112.2 ± 2.81	0.286 ± 0.032	17.5 ± 0.49	27.08 ± 5.19
MT-CLPs-0.50	171.1 ± 2.01	0.297 ± 0.004	25.3 ± 0.97	27.76 ± 3.04
MT-CLPs-1.0	265.4 ± 4.32	0.312 ± 0.022	33.9 ± 2.86	22.05 ± 2.44

**Table 2:** In vitro release kinetics models of MT-LPs, MT-CLPs-0.25, and MT-CLPs-0.5 (*n* = 3, mean ± SD).

Correlation Coefficient (R^2^)					
Formulations	Zero-Order	First-Order	Higuchi’s Model	Korsmeyer–Peppas Model	
	R^2^				*n*
MT-LPs	0.842 ± 0.086	0.916 ± 0.113	0.932 ± 0.038	0.969 ± 0.014	0.201 ± 0.024
MT-CLPs-0.25	0.895 ± 0.076	0.983 ± 0.055	0.975 ± 0.033	0.987 ± 0.016	0.362 ± 0.064
MT-CLPs-0.50	0.799 ± 0.047	0.908 ± 0.059	0.925 ± 0.028	0.963 ± 0.009	0.318 ± 0.032

**Table 3:** Parameters of the in vivo effectiveness post-instillation of 0.5 % (*w/v*) MT of different formulations into rabbit eyes (*n* = 3, mean ± SD).

PD Parameters	MT-ISG	MT-ISG1	MT-ISG2
(% Dec IOP)_max_ (%)	54.7 ± 3.15	62.3 ± 6.28	73.6 ± 5.13
t_max_ (h)	4	4	3
AUC_(0–6)_ (%.h)	199.2 ± 11.73	256.5 ± 19.26	279.1 ± 27.83
MRT (h)	2.3 ± 0.91	5.7 ± 0.82	6.2 ± 1.19

**Table 4:** Scores obtained in Draize’s test of various formulations in rabbit eyes.

Formulations	1 h	3 h	6 h	24 h
MT-ISG	0	0	0	0
MT-ISG1	0	0	0	0
MT-ISG2	(Conjunctival redness)	0	0	0

## Data Availability

Not applicable.
